# Left ventricular stroke volume index following transcatheter aortic valve replacement is an early predictor of 1‐year survival

**DOI:** 10.1002/clc.23937

**Published:** 2022-10-23

**Authors:** Shiva K. Annamalai, Benjamin C. Koethe, Eli Simsolo, Dou Huang, Ann Connors, Charles D. Resor, Andrew R. Weintraub, Natesa G. Pandian, Brian C. Downey, Ayan R. Patel, Benjamin S. Wessler

**Affiliations:** ^1^ The CardioVascular Center Tufts Medical Center Boston Massachusetts USA; ^2^ Department of Medicine Tufts Medical Center Boston Massachusetts USA

**Keywords:** echocardiography, stroke volume index, TAVR

## Abstract

**Background:**

Adverse cardiac events are common following transcatheter aortic valve replacement (TAVR). Our aim was to investigate the low left ventricular stroke volume index (LVSVI) 30 days after TAVR as an early echocardiographic marker of survival.

**Hypothesis:**

Steady‐state (30‐day) LVSVI after TAVR is associated with 1‐year mortality.

**Methods:**

A single‐center retrospective analysis of all patients undergoing TAVR from 2017 to 2019. Baseline and 30‐day post‐TAVR echocardiographic LVSVI were calculated. Patients were stratified by pre‐TAVR transaortic gradient, surgical risk, and change in transvalvular flow following TAVR.

**Results:**

This analysis focuses on 238 patients treated with TAVR. The 1‐year mortality rate was 9% and 124 (52%) patients had normal flow post‐TAVR. Of those with pre‐TAVR low flow, 67% of patients did not normalize LVSVI at 30 days. The 30‐day normal flow was associated with lower 1‐year mortality when compared to low flow (4% vs. 14%, *p* = .007). This association remained significant after adjusting for known predictors of risk (adjusted odds ratio [OR] of 3.45, 95% confidence interval: 1.02–11.63 [per 1 ml/m^2^ decrease], *p* = .046). Normalized transvalvular flow following TAVR was associated with reduced mortality (8%) when compared to those with persistent (15%) or new‐onset low flow (12%) (*p* = .01).

**Conclusions:**

LVSVI at 30 days following TAVR is an early echocardiographic predictor of 1‐year mortality and identifies patients with worse intermediate outcomes. More work is needed to understand if this short‐term imaging marker might represent a novel therapeutic target.

**Abbreviations**
AS: aortic stenosis
LF: low flow
LV: left ventricle
LVEF: left ventricular ejection fraction
LVOT: left ventricular outflow tract
LVOT_D_
: left ventricular outflow tract diameter
LVOT_VTI_
: left ventricular outflow tract velocity time integral
LVSVI: left ventricular stroke volume index
NF: normal flow
SAVR: surgical aortic valve replacement
TAVR: transcatheter aortic valve replacement
TTE: transthoracic echocardiogram

## 1 INTRODUCTION

Transcatheter aortic valve replacement (TAVR) has become the treatment of choice for patients older than 65 with severe symptomatic aortic stenosis (AS), regardless of their predicted risk.^1^ However, despite technological advances and increased operator experience, a substantial minority of patients still experience poor outcomes, including death, poor quality of life, or quality of life decline. Preprocedure hemodynamics, such as low left ventricle stroke volume index (LVSVI) and left ventricular ejection fraction (LVEF), portend worse outcomes after TAVR.^2^, ^3^, ^4^ The hazards associated with these markers are modest, and despite multivariate modeling, our ability to identify patients in advance of treatment who are unlikely to benefit from TAVR remains limited.^5^ Ultimately, most symptomatic patients are treated with TAVR because of the potential for clinical improvement—a trend that has led to very high utilization of TAVR.^6^

There is an important gap in our ability to identify patients early after TAVR who are unlikely to do well. Short‐term postprocedure predictors of longer‐term outcomes are necessary to align patient and family expectations with anticipated outcomes and ideally to target additional interventions to those least likely to do well. In a post hoc analysis of trial patients with low‐flow (LF) AS, persistent LF on transthoracic echocardiogram (TTE) after TAVR but before discharge was associated with increased 1‐year mortality.^7^ Imaging done immediately after TAVR (before discharge) does not capture steady‐state hemodynamics and whether or not a relationship between a steady‐state postprocedural LVSVI and intermediate‐term mortality exists for contemporary unselected patients remains unknown. Here, we evaluate LF, defined as LVSVI 30 days after TAVR as an early and easily assessed echocardiographic marker of intermediate‐term survival for unselected patients following TAVR.

## 2 MATERIALS AND METHODS

A single‐center retrospective analysis of all patients with severe AS undergoing TAVR between 2017 and 2019 was performed. This study was approved by the Tufts Medical Center Institutional Review Board. No informed consent was required because this was a retrospective study. Baseline demographics and 1‐year mortality were collected through a review of the Institutional Society of Thoracic Surgeons/American College of Cardiology Transcatheter Valve Therapy (STS/ACC TVT) Registry report. Baseline and 30‐day postprocedural TTE data were collected. For this study, preprocedure and postprocedure left ventricular outflow tract (LVOT) diameter (LVOT_D_) and Doppler velocity time integrals (LVOT_VTI_) were measured by blinded members of the study team. LVOT_D_ and LVOT_VTI_ were collected consistent with the current guidelines.^8^ LVOT_VTI_ was averaged over three cardiac cycles for patients in sinus rhythm and five cycles for patients in atrial fibrillation. Before TAVR, LVOT_D_ was measured inner edge to inner edge, parallel and adjacent to the aortic valve during mid‐systole from the two‐dimensional parasternal long‐axis view, whereas after TAVR, LVOT_D_ was measured just proximal to the apical border of the stent as was done in the pivotal clinical trials. The LVOT_VTI_ was obtained by tracing the modal velocity of a pulsed‐wave Doppler assessment from the apical long‐axis or five‐chamber view with the sample volume positioned just proximal to the prevalve acceleration region. One investigator (S. K. A.) collected all LVOT_D_ measurements. Two investigators (E. S. and D. H.) collected all LVOT_VTI_ measurements; an initial 10% sample was measured in duplicate by the two investigators to ensure standardization of technique. These tracings were independently reviewed by a board‐certified echocardiographer (B. S. W.). A priori the decision was made to do single investigator tracings of the remaining data when an average discrepancy of <5% was obtained. LVOT‐derived LVSVI was subsequently calculated at both time points as ${\mathrm{LVOT}}_{\mathrm{VTI}}\times \pi {\left(\frac{{\mathrm{LVOT}}_{D}}{2}\right)}^{2}$. LF is defined as LVSVI < 35 ml/m^2^ and normal flow (NF) is defined as LVSVI ≥ 35 ml/m^2^.

## 3 STATISTICAL ANALYSIS

We examined the relationship between LVSVI at 30 days and 1‐year survival using logistic regression. A complete case analysis was used. Normal LVSVI was defined as >35 ml/m^2^. Multivariable regression was performed to adjust for 30‐day LVEF, age, sex, height, weight, presence of hypertension, diabetes, atrial fibrillation/atrial flutter, creatinine, prior coronary artery bypass grafting (CABG) prior stroke, presence of peripheral vascular disease, smoking status, and STS score. The intent of this modeling exercise was not to create a predictive model, but instead to see if the effect of LVSVI was independent of these known outcome predictors. As exploratory analyses, patients were categorized by pre‐TAVR gradient (normal gradient [NG] and low gradient [LG], defined by mean transaortic gradient greater than or less than 40 mm Hg, respectively) and operator risk (intermediate vs. high/extreme risk), as well as by changes in transvalvular flow from baseline to the 30‐day post‐TAVR (persistent LF [baseline: LF; post‐TAVR: LF]; normalized flow [baseline: LF; post‐TAVR: NF]; new‐onset LF [baseline: NF; post‐TAVR: LF]; maintained NF [baseline: NF; post‐TAVR: NF]) and a similar subset analysis was performed. Operator risk in this study was defined by the operator in a standard fashion integrating STS score and procedural factors.

## 4 RESULTS

**Figure 1:**
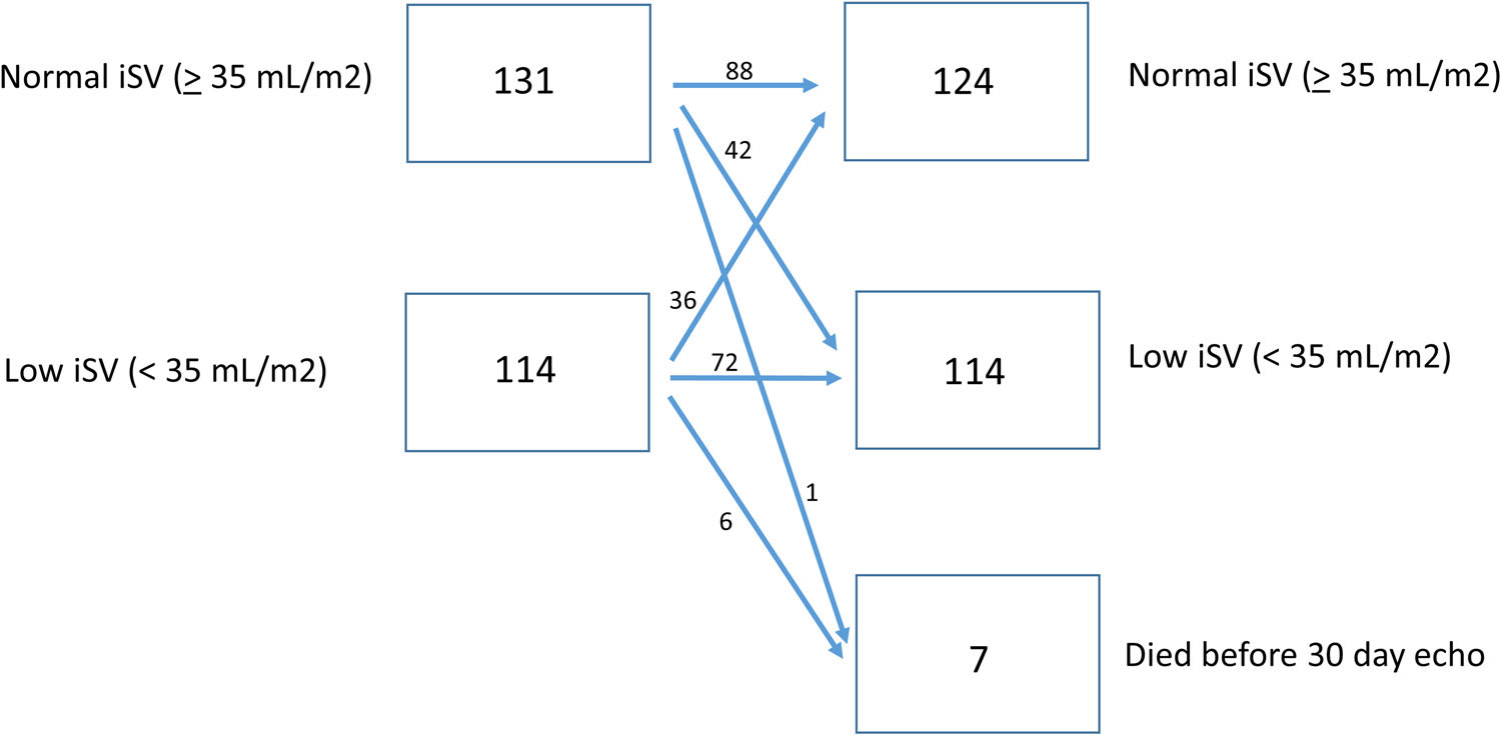
Reclassification of indexed LVOT‐derived stroke volume after TAVR. The primary analytic cohort includes patients with pre‐ and 30‐day postechocardiograms (*N* = 238). iSV, indexed stroke volume; LVOT, left ventricular outflow tract; ml, milliliter, m^2^, meters squared; TAVR, transcatheter aortic valve replacement.

A total of 279 patients were treated with TAVR during this timeframe. Seven patients (2%) died before 30‐day follow‐up (Supporting Information: Table 1). This analysis focuses on 238 patients (85%) with preprocedure and 30‐day follow‐up TTE and known vital status at 1 year (Figure 1).

### 4.1 Preprocedure and postprocedure characteristics

Baseline demographics and echocardiographic data are presented in Table 1. For the total cohort, baseline characteristics included an average age of 81 ± 8 years, 55% male, average STS score of 6.9 ± 5.2, 45% intermediate surgical risk, 55% high/extreme surgical risk, average LVEF 55 ± 11%, average LVSVI 37 ± 9 ml/m^2^, and average AVA 0.8 ± 0.2 cm^2^. One hundred and forty‐one (59%) patients had pre‐TAVR LG and 108 (45%) patients had pre‐TAVR low‐flow.

**Table 1:** Baseline demographics and echocardiographic findings for patients with 30‐day LVSVI < 35 versus ≥35 ml/m^2^

Baseline demographics	LVSVI (ml/m^2^)			
	Total	<35	≥35	*p* Value
Patients	238.85%	114.48%	124.52%	
Age (years)	81 ± 8	80 ± 8	82 ± 8	.79
Sex (male, %)	132.55%	75.66%	57.46%	.002
BSA (m^2^)	1.9 ± 0.3	2 ± 0.2	1.8 ± 0.3	.99
BMI (kg/m^2^)	28.8 ± 9.3	29.2 ± 5.4	28.5 ± 11.9	.83
Hypertension	206. 87%	99.87%	107.86%	.99
Diabetes	79.33%	46.40%	33.27%	.03
Atrial fibrillation	90.38%	51.45%	39.31%	.04
Creatinine (mg/dl)	1.3 ± 1	1.2 ± 0.6	1.3 ± 1.3	.97
Prior PCI	51.21%	26.23%	25.20%	.64
Prior MI	39.16%	29.25%	10.8%	<.001
Prior CABG	37.16%	21.18%	16.13%	.28
Prior stroke	29.12%	11.10%	18, 15%	.32
Prior PAD	57.24%	23.20%	34.27%	.22
Home O_2_	18.8%	11.10%	7.6%	.33
Smoker	9, 4%	3, 3%	6, 5%	.5
NYHA III or IV	187.79%	91.80%	96.77%	.75
STS risk score	6.9 ± 5.2	7.3 ± 6	6.5 ± 4.2	.88
Intermediate risk	108.45%	52.46%	56.45%	.99

Baseline echocardiographic findings	LVSVI (ml/m^2^)			
	Total	<35	≥35	*p* Value
LVEF (%)	55 ± 11	53 ± 12	57 ± 11	.05
LVSVi (ml/m^2^)	37 ± 9	33 ± 6	40 ± 10	<.001
LVIDd (cm)	4.3 ± 0.8	4.4 ± 0.9	4.3 ± 0.7	.98
Aortic valve characteristics				
Bicuspid	15.6%	6.5%	9.7%	.6
AVA (cm^2^)	0.8 ± 0.2	0.8 ± 0.2	0.8 ± 0.2	.99
Mean gradient (mm Hg)	38 ± 14	37 ± 13	39 ± 14	.76
Peak velocity (m/s)	4 ± 0.9	4.1 ± 1	4.1 ± 0.7	.99
Concomitant valve disease				
AI Mod or severe	29.12%	12.11%	17.14%	.55
MS	38.16%	15.13%	23.19%	.29
MR Mod or severe	33.14%	18.16%	15.12%	.46
TR Mod or severe	25.11%	17.15%	8.6%	.04

Abbreviations: AI Mod, aortic insufficiency moderate; AVA, aortic valve area; BMI, body mass index; BSA, bovine serum albumin; CABG, coronary artery bypass grafting; LVEF, left ventricular ejection fraction; LVIDd, left ventricle internal diameter during diastole; LVOT, left ventricular outflow tract; LVOT_D_, LVOT diameter; LVOT_VTI_, LVOT Doppler velocity time integral; LVSVI, left ventricular stroke volume index; MI, myocardial infarction; MR Mod, mitral regurgitation moderate; NYHA, New York Heart Association; PAD, peripheral artery disease; PCI, percutaneous coronary intervention; STS, Society of Thoracic Surgeons; TR Mod, tricuspid regurgitation moderate.

Similar rates of nontransfemoral access occurred in each group (LF: 13% [transaxillary: 9%; transapical: 4%] versus NF: 9% [transaxillary: 3%; transapical: 5%; transaortic: 1%], *p* = .31). Postprocedure, the average LVEF was 55 ± 11% and the average LVSVI was 37 ± 10 ml/m^2^.

### 4.2 Postprocedure flow

**Figure 2:**
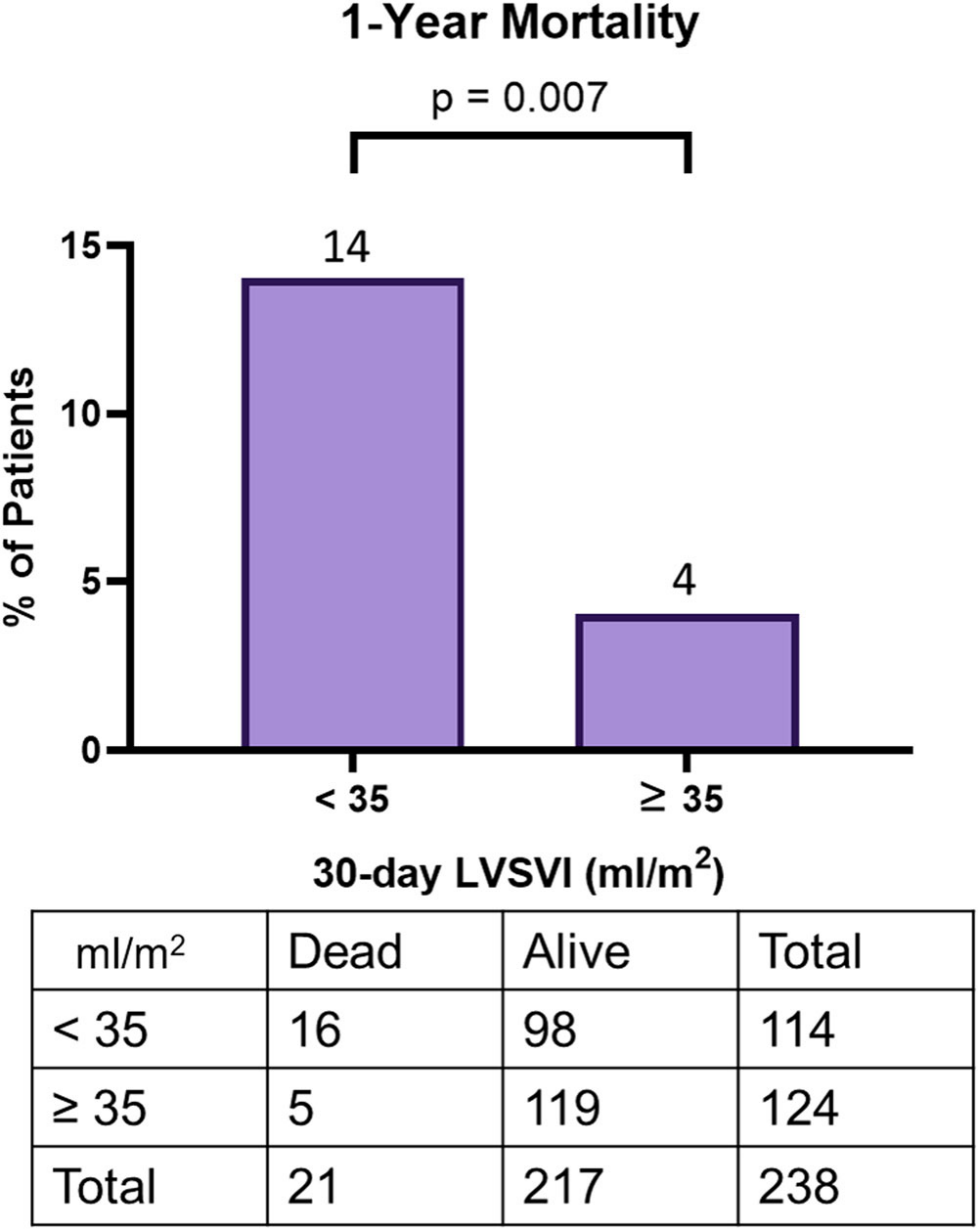
One‐year mortality among patients undergoing transcatheter aortic valve replacement when dichotomized by 30‐day follow‐up left ventricular stroke volume index (LVSVI) < or ≥35 ml/m^2^

The postprocedure TTE was done an average of 32 ± 6 days after TAVR. At 30 days, 114 (48%) patients had LF, and 124 (52%) patients had NF. Average 30‐day LVSVI was 29 ± 5 and 44 ± 7 ml/m^2^, for 30‐day LF and NF, respectively. Thirty‐day LF was associated with increased 1‐year mortality when compared to NF (14% vs. 4%, *p* = .007) (Figure 2). Thirty‐day LF remained associated with increased 1‐year mortality after adjusting for 30‐day LVEF, age, sex, height, weight, presence of hypertension, diabetes, atrial fibrillation/atrial flutter, creatinine, prior CABG, prior stroke, presence of peripheral vascular disease, smoking status and STS score (odds ratio [OR] of 3.45, 95% confidence interval [CI]: 1.02–11.63 [per 1 ml/m^2^ increase], *p* = .046) (Supporting Information: Table 2).

### 4.3 Relationship with pre‐TAVR transaortic gradient

**Figure 3:**
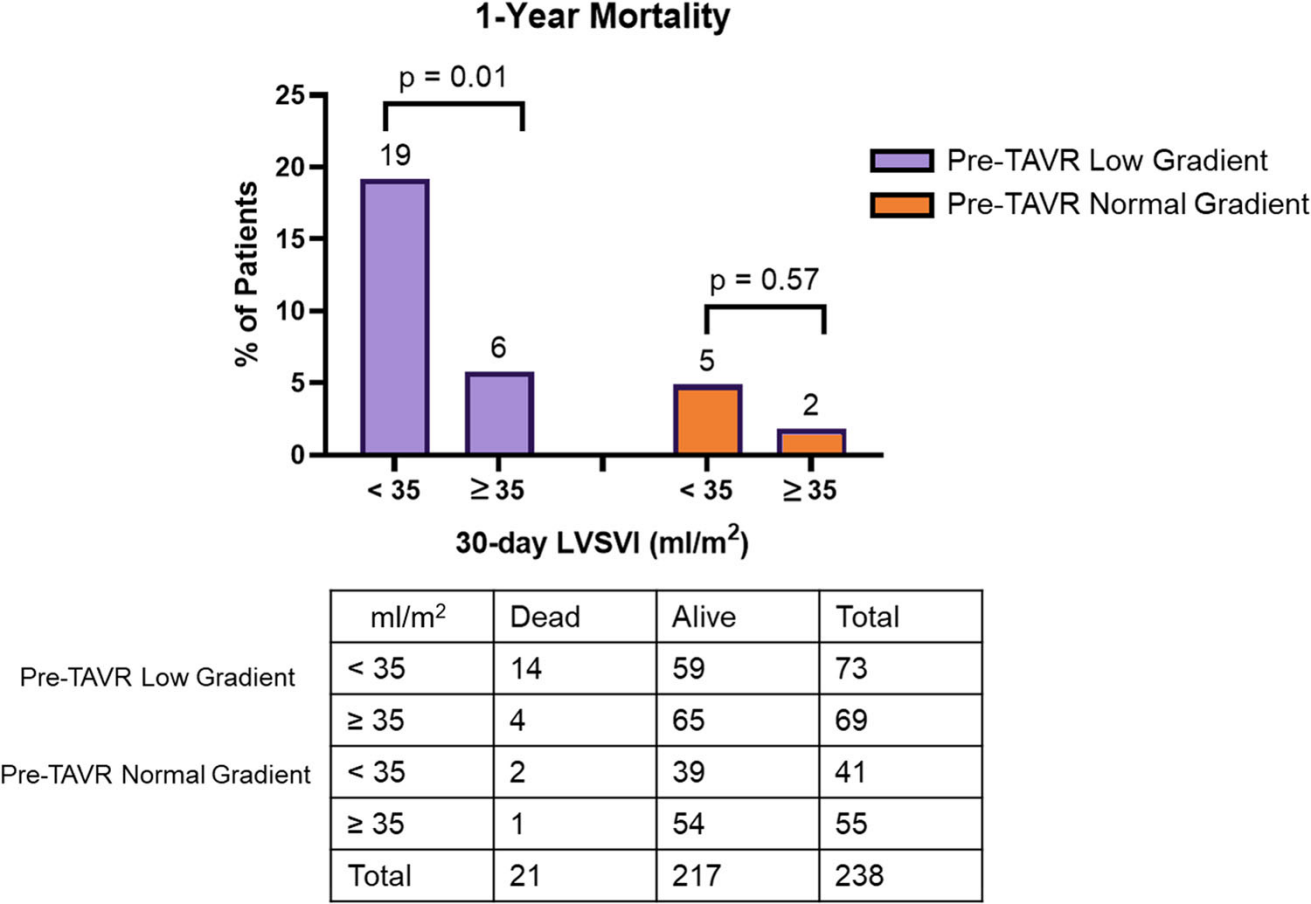
One‐year mortality among patients undergoing transcatheter aortic valve replacement when stratified by both 30‐day follow‐up LVSVI < or ≥35 ml/m^2^, as well as preprocedural transvalvular gradient. LVSVI, left ventricular stroke volume index; TAVR, transcatheter aortic valve replacement.

A total of 142 (60%) patients had pre‐TAVR LG severe AS and 96 (40%) patients had pre‐TAVR NG severe AS. The average baseline mean gradient was 29 ± 7 and 51 ± 19 mm Hg, respectively, in these two groups. Thirty‐day LF was associated with increased 1‐year mortality in those with pre‐TAVR LG (LF vs. NF: 19% vs. 6%, *p* = .01). There was no association between postprocedure flow and mortality for patients with preop NG severe AS. In this subset, 30‐day NF and LF had 1‐year mortality of 2% and 5% (*p* = .57), respectively (Figure 3).

### 4.4 Relationship with pre‐TAVR surgical risk

Pre‐TAVR, 108 (45%) patients had intermediate surgical risk and 130 (55%) patients had high or extreme risk. Thirty‐day LF was associated with increased 1‐year mortality in those with intermediate risk (LF vs. NF: 10% vs. 0%, *p* = .02). There was a trend toward increased 1‐year mortality with 30‐day LF in those with high or extreme risk (LF vs. NF: 18% vs. 7%, *p* = .07) (Supporting Information: Figure 1).

### 4.5 Relationship with transvalvular flow changes

**Figure 4:**
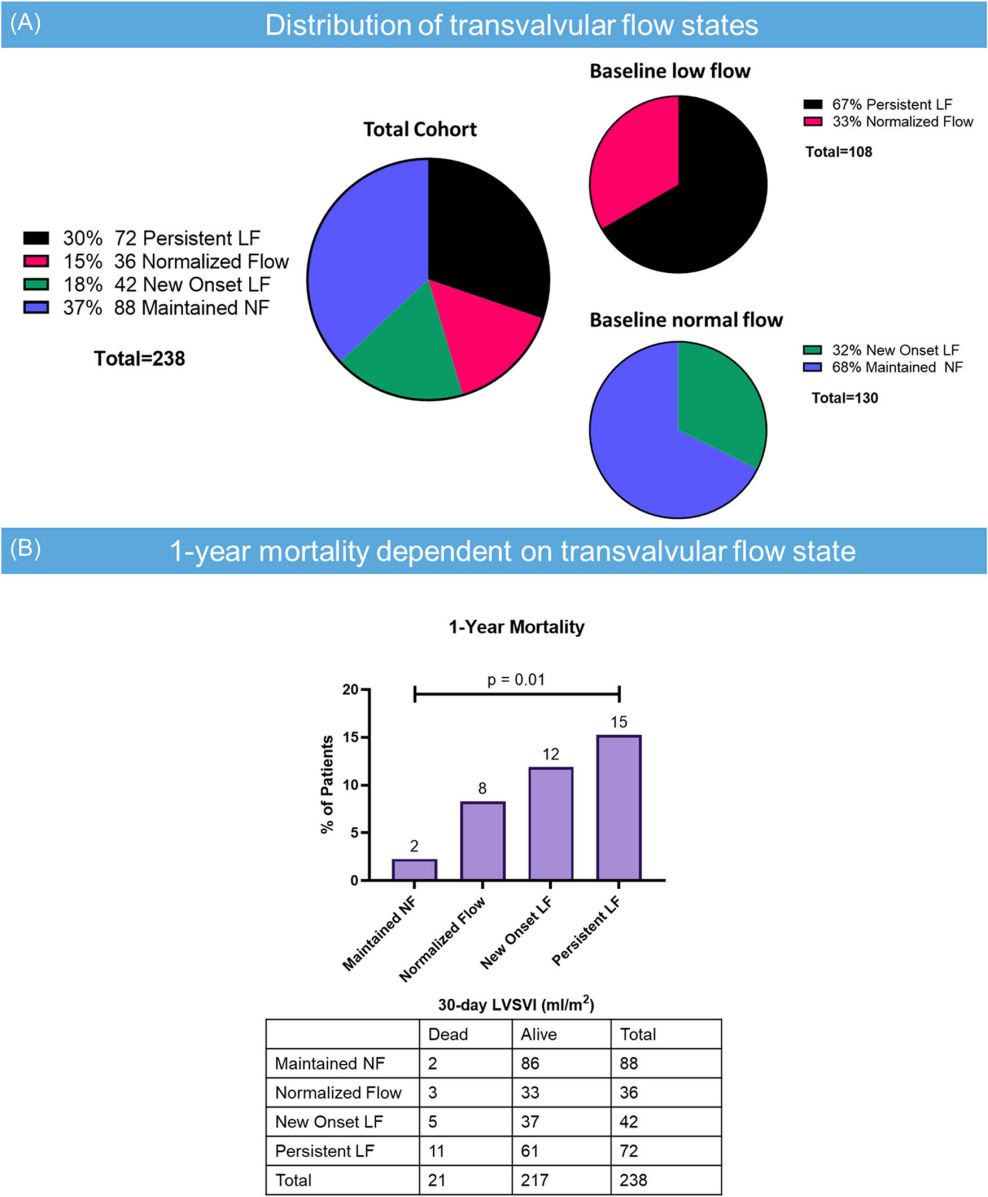
(A) Distributions of transvalvular flow states following a transcatheter aortic valve replacement. (B) Relationship between transvalvular flow states and 1‐year mortality. LF, low flow, NF, normal flow.

When comparing flow measurements pre‐ and post‐TAVR, it is important to consider the different standards for measuring LVOTd (inner edge to inner edge vs. just proximal to the apical border of the stent. Here, the LVOT was measured larger before TAVR (43%), larger following TAVR (18%), and no change (39%) following TAVR; 72 (30%) patients had persistent LF, 35 (15%) patients had normalized flow, 42 (18%) had new‐onset LF, and 88 (42%) had maintained NF. Among those with pre‐TAVR LF, 67% had continued LF following TAVR. Among those with pre‐TAVR NF, 68% had continued NF following TAVR (Figure 4A). Baseline demographics and echocardiographic data are presented in Supporting Information: Table 3. For the patients with new onset of LF after TAVR, many patients had additional valve disease or diastolic dysfunction that persisted at 30 days (Supporting Information: Table 4). Nine percent of patients with new‐onset LF had significant TR, 7% had significant MS, and 36% had abnormalities of diastolic function. One‐year mortality was associated with the change in transvalvular flow after TAVR (*p* = .01 for trend; Figure 4B).

## 5 DISCUSSION

This study represents the first analysis of short‐term steady‐state LVSVI as a predictor of intermediate‐term outcomes in unselected patients with intermediate or greater surgical risk undergoing TAVR with contemporary care. The key findings from this report are that approximately 2/3 of patients with low‐preprocedural flow do not increase their LVSVI after TAVR and that LF defined as echocardiographic derived LVSVI < 35 ml/m^2^ at 30 days post‐TAVR is an early and routinely assessed imaging marker that is associated with increased 1‐year postprocedural mortality. Those with persistent or new‐onset LF postprocedure had worse 1‐year outcomes following TAVR. This imaging finding can be used early after TAVR to identify patients unlikely to do well.

These results extend earlier work showing that immediate postprocedure LVSVI was associated with worse outcomes in an earlier therapeutic era of high‐ or extreme‐risk TAVR patients.^9^ Since that time, there has been continued operator experience and device iterations resulting in progressively declining rates of postprocedural mortality, major vascular complications, permanent pacemaker implantation, and moderate or greater paravalvular leak.^10^, ^11^, ^12^ The associations between postprocedure flow and outcomes persist in the setting of improved procedural success and the treatment of lower‐risk patients. Our analysis focuses for the first time on 30‐day post‐TAVR TTE, which guidelines recommend as timing for postprocedural imaging to achieve normal loading conditions compared to early shifted hemodynamics.^1^ As future clinical trials are designed to study lower‐risk patients who are earlier in the disease course, this imaging marker can be considered as part of a composite endpoint that might improve the ability to detect meaningful changes.

In our analysis, roughly 1/3 of patients with preprocedural LF increased their flow and 1/3 of patients with preprocedural NF decreased their flow. These findings should be interpreted cautiously and need to be replicated since they may be at least partially related to minor changes in measured LVOTd pre‐ and post‐TAVR. Since almost all symptomatic AS patients are offered TAVR, our findings suggest that postprocedure flow may be an important marker of intermediate‐term outcomes after a technically successful procedure. A drop in transvalvular flow following TAVR may result from a number of causes. Many potential causes of new‐onset LF have been proposed, including acute unmasking of underlying preprocedural comorbid conditions, concomitant valvular disease, periprocedural complications, such as new or worsened systolic dysfunction, new atrial fibrillation, major bleeding, or patient–prosthesis mismatch, resulting in altered hemodynamics.^13^, ^14^ Our analysis of these patients shows that persistent tricuspid regurgitation and mitral valve disease are common and that over 1/3 of these patients have evidence of diastolic abnormalities. Each of these abnormalities may represent a future therapeutic target.

Noninvasive SV based on an assumed circular LVOT configuration was first described by Lewis et al.^15^ in 1984 and correlated well with thermodilution estimates of stroke volume and cardiac output. This measurement is more challenging in the setting of significant calcium associated with severe AS and it is now well‐appreciated that the circular LVOT assumption is often violated.^8^, ^16^ Recognizing the limitations of this measurement, it remains a routinely assessed imaging parameter for patients with AS. Those starting with low transvalvular flow likely represent a heterogeneous group of patients^17^ and those who normalize their flow likely have fewer of these conditions, whereby reduced afterload following TAVR combined with contractile reserve allows for an increased transvalvular flow. In this study, persistent LF patients trended toward the increased prevalence of diabetes and myocardial infarction, as well as more tricuspid regurgitation, which itself has been previously shown to be more prevalent in patients with paradoxical LF LG AS with persistent LF following TAVR.^18^ These associated conditions may be related to the higher mortality rates for this cohort, although additional studies are needed.

Limitations of this report primarily relate to its retrospective nature. However, the echocardiographic data were meticulously extracted in a blinded fashion consistent with current guidelines. Echocardiographically derived LVSVI may be an underestimate of the true LVSVI due to an underestimated LVOT area, given the elliptical shape of the anatomic LVOT.^8^ LVOT area could be more reliably assessed using LVOT area obtained during CTA imaging, although this integrative imaging approach would not be easily accessible at follow‐up after TAVR. Certainly, these results should be reproduced with more accurate LVOT area assessments. However, the relationship between 30‐day echocardiographically derived LVSVI and mortality following TAVR provides a more routinely available data point than utilizing computed tomography‐derived LVOT that would require additional (not routine) postprocedure testing. There is inherent survival bias in our study as our analysis is dependent on those who survived the 30‐day postprocedural TTE. Only seven patients (3%) died before 30‐day follow‐up. These findings suggest that our analysis demonstrates a conservative estimate of the significance of postprocedural flow when considering these patients who died before 30 days.

LVSVI at 30 days represents a routinely measured echocardiographic variable that is associated with worse outcomes after TAVR. More work is needed to understand if this marker represents a novel therapeutic target that might be modifiable and a target of future interventions.

## 6 CONCLUSIONS

In an unselected real‐world TAVR‐treated cohort with intermediate and high or extreme operative risk, LVSVI at 30 days following TAVR is an early echocardiographic predictor of 1‐year mortality. As utilization of TAVR continues to rise and fewer patients receive medical therapy, this imaging finding may help identify patients early after TAVR who are at risk for worse 1‐year survival and for which additional interventions to improve LVSVI should be considered.

## Supporting information

Supporting information.

Supporting information.

## Data Availability

The data that support the findings of this study are available on request from the corresponding author. The data are not publicly available due to privacy or ethical restrictions.
