# Supplementary figures and images for: Left ventricular stroke volume index following transcatheter aortic valve replacement is an early predictor of 1‐year survival
Source: Clin Cardiol. 2022 Oct 23;46(1):76–83. doi: 10.1002/clc.23937 (PMC9849436; doi:10.1002/clc.23937)

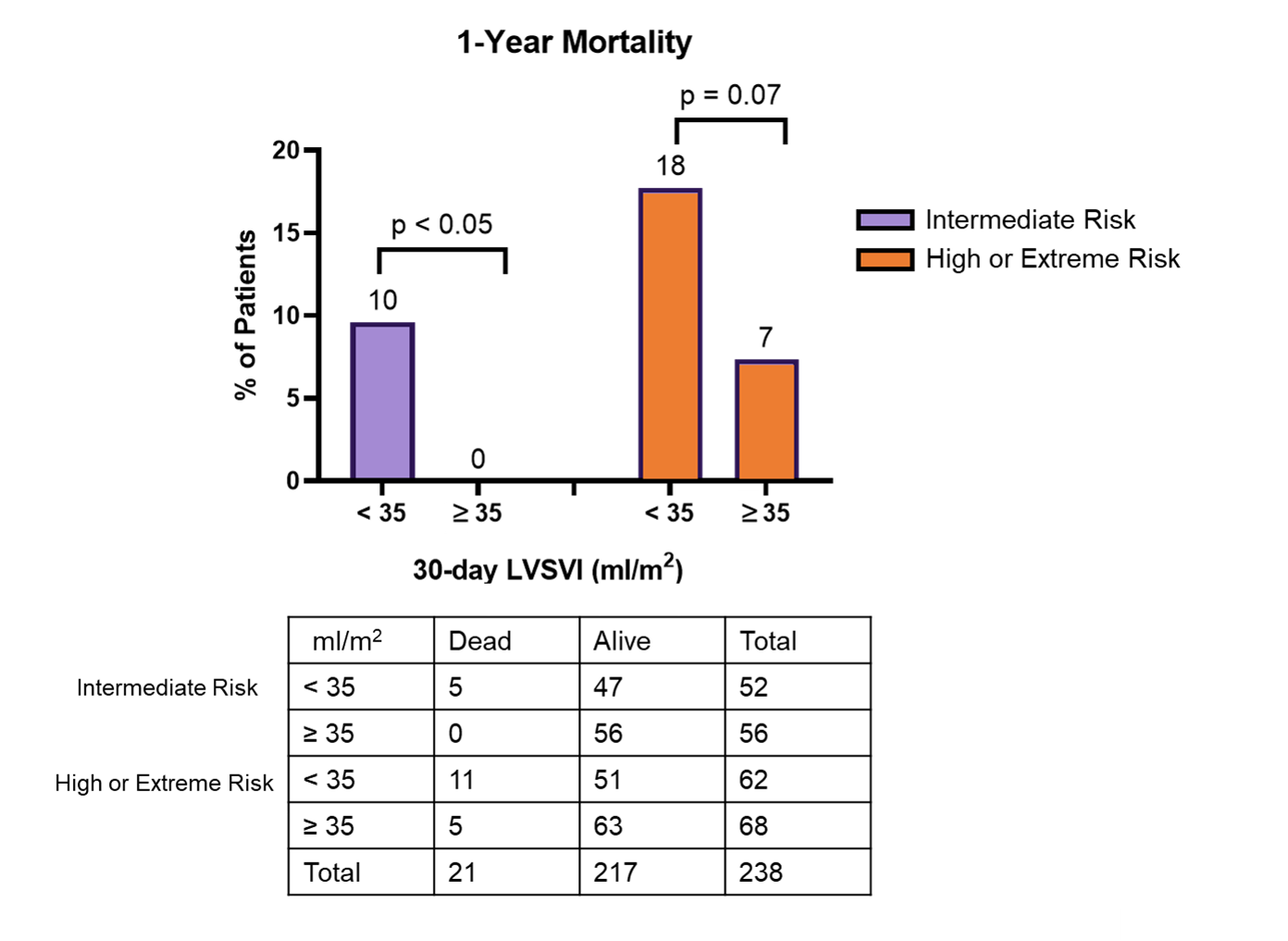

Supplement: Supplementary file 1 — Supporting information. [file CLC-46-76-s002.tif]
