# Supplementary material for: Left ventricular stroke volume index following transcatheter aortic valve replacement is an early predictor of 1‐year survival
Source: Clin Cardiol. 2022 Oct 23;46(1):76–83. doi: 10.1002/clc.23937 (PMC9849436; doi:10.1002/clc.23937)
Supplement: Supplementary file 2 — Supporting information. [file CLC-46-76-s001.docx]

Supplemental Tables:

Supplemental Table 1: Baseline demographics and echocardiographic findings for patients who died prior to 30-days.

| **Baseline Demographics** | |
| --- | --- |
|  | Death prior to 30 days |
| **Patients** | 7, 3% |
| **Age (yrs)** | 84 ± 8 |
| **Sex (Male, %)** | 5, 71% |
| **BSA (m2)** | 1.9 ± 0.2 |
| **BMI (kg/m2)** | 25.8 ± 5.3 |
| **Hypertension** | 5, 71% |
| **Diabetes** | 4, 57% |
| **Atrial Fibrillation** | 4, 57% |
| **Creatinine (mg/dL)** | 1.4 ± 0.5 |
| **Prior PCI** | 1, 14% |
| **Prior MI** | 1, 14% |
| **Prior CABG** | 2, 29% |
| **Prior Stroke** | 1, 14% |
| **Prior PAD** | 2, 29% |
| **Home O2** | 1, 14% |
| **Smoker** | 0, 0% |
| **NYHA III or IV** | 6, 86% |
| **STS Risk Score** | 14.6 ± 7.4 |
| **Intermediate Risk** | 1, 14% |
| **Baseline Echocardiographic Findings** | |
|  | Death prior to 30 days |
| **LVEF (%)** | 34 ± 18 |
| **LVSVi (ml/m2)** | 29 ± 14 |
| **LVIDd (cm)** | 3.8 ± 0.8 |
| **Aortic Valve Characteristics** | |
| **Bicuspid** | 0, 0% |
| **AVA (cm2)** | 0.7 ± 0.3 |
| **Mean gradient (mmHg)** | 37 ± 19 |
| **Peak Velocity (m/s)** | 5.2 ± 1 |
| **Concomitant Valve Disease** | |
| **AI Mod or Severe** | 0, 0% |
| **MS** | 1, 14% |
| **MR Mod or Severe** | 5, 71% |
| **TR Mod or Severe** | 2, 29% |

Supplemental Table 2. Multivariable Logistic Regression for 1 year Mortality after TAVR

| Odds Ratio Estimates | | | | |
| --- | --- | --- | --- | --- |
| Effect | Point Estimate | 95% Wald | | P-Value |
|  |  | Confidence Limits | |  |
| **SVI_30d < 35** | **3.45** | **1.02** | **11.63** | **0.046** |
| 30 Day LVEF | 0.98 | 0.93 | 1.02 | 0.286 |
| Age | 1.05 | 0.97 | 1.14 | 0.251 |
| Male | 0.18 | 0.03 | 0.96 | 0.044 |
| Height | 1.06 | 0.98 | 1.15 | 0.129 |
| Weight | 0.99 | 0.95 | 1.03 | 0.547 |
| Hypertension | 2.70 | 0.31 | 23.55 | 0.368 |
| Diabetes | 1.38 | 0.46 | 4.15 | 0.570 |
| Afib/Flutter | 2.44 | 0.82 | 7.27 | 0.109 |
| Cr | 1.08 | 0.54 | 2.15 | 0.835 |
| Prior CABG | 3.07 | 0.83 | 11.31 | 0.093 |
| Prior Stroke | 0.84 | 0.15 | 4.52 | 0.835 |
| Prior PAD | 0.88 | 0.26 | 3.06 | 0.846 |
| Smoker | 4.66 | 0.38 | 57.45 | 0.230 |
| STS Score | 1.00 | 0.90 | 1.11 | 0.935 |

Supplemental Table 3: Baseline demographics and echocardiographic findings dependent on transvalvular flow state following TAVR.

| **Baseline Demographics** | | |  |  |  |  |  |
| --- | --- | --- | --- | --- | --- | --- | --- |
|  | Pre-LVSVI ≥ 35 ml/m2 | | |  | Pre-LVSVI < 35 ml/m2 | | |
|  | Maintained NF | New Onset LF | p-value |  | Normalized Flow | Persistent LF | p-value |
| **Patients** | 88, 37% | 42, 18% |  |  | 36, 15% | 72, 30% |  |
| **Age (yrs)** | 82 ± 8 | 81 ± 9 | 0.96 |  | 81 ± 8 | 80 ± 7 | 0.75 |
| **Sex (Male, %)** | 34, 39% | 28, 67% | 0.01 |  | 23, 64% | 47, 65% | 0.89 |
| **BSA** | 1.8 ± 0.3 | 1.9 ± 0.2 | 0.98 |  | 2 ± 0.2 | 2 ± 0.2 | 0.99 |
| **BMI** | 28 ± 13.4 | 28.2 ± 5.7 | 0.99 |  | 29.7 ± 6.7 | 29.7 ± 5.1 | 0.99 |
| **Hypertension** | 76, 86% | 35, 83% | 0.65 |  | 31, 86% | 64, 89% | 0.68 |
| **Diabetes** | 21, 24% | 11, 26% | 0.77 |  | 12, 33% | 35, 49% | 0.13 |
| **Atrial Fibrillation** | 22, 25% | 15, 36% | 0.22 |  | 17, 47% | 36, 50% | 0.79 |
| **Creatinine** | 1.4 ± 1.5 | 1.2 ± 0.5 | 0.46 |  | 1.1 ± 0.2 | 1.3 ± 0.7 | 0.76 |
| **Prior PCI** | 17, 19% | 6, 14% | 0.48 |  | 8, 22% | 20, 28% | 0.51 |
| **Prior MI** | 5, 6% | 9, 21% | 0.007 |  | 5, 14% | 20, 28% | 0.11 |
| **Prior CABG** | 10, 11% | 6, 14% | 0.64 |  | 6, 17% | 15, 21% | 0.61 |
| **Prior Stroke** | 10, 11% | 4, 10% | 0.75 |  | 8, 22% | 7, 10% | 0.08 |
| **Prior PAD** | 17, 19% | 5, 12% | 0.29 |  | 17, 47% | 18, 25% | 0.02 |
| **Home O2** | 6, 7% | 3, 7% | 0.95 |  | 1, 3% | 8, 11% | 0.14 |
| **Smoker** | 4, 5% | 1, 2% | 0.55 |  | 2, 6% | 2, 3% | 0.47 |
| **NYHA III or IV** | 66, 75% | 33, 79% | 0.66 |  | 30, 83% | 58, 81% | 0.73 |
| **STS Risk Score** | 6.8 ± 4.3 | 6.9 ± 5.5 | 0.99 |  | 6 ± 4 | 7.5 ± 6.3 | 0.47 |
| **Intermediate Risk** | 36, 41% | 20, 56% | 0.53 |  | 19, 45% | 33, 46% | 0.34 |
| **Baseline Echocardiographic Findings** | | | |  |  |  |  |
|  | Pre-LVSVI ≥ 35 ml/m2 | | |  | Pre-LVSVI < 35 ml/m2 | | |
|  | Maintained NF | New Onset LF | p-value |  | Normalized Flow | Persistent LF | p-value |
| **LVEF (%)** | 59 ± 9 | 56 ± 11 | 0.71 |  | 53 ± 13 | 51 ± 12 | 0.77 |
| **LVSVi (ml/m2)** | 45 ± 7 | 39 ± 4 | <0.0001 |  | 29 ± 5 | 29 ± 4 | 0.99 |
| **LVIDd (cm)** | 4.2 ± 0.6 | 4.4 ± 0.7 | 0.44 |  | 4.5 ± 0.7 | 4.4 ± 0.9 | 0.62 |
| **Aortic Valve Characteristics** | | |  |  |  |  |  |
| **Bicuspid** | 6, 7% | 2, 5% | 0.04 |  | 3, 8% | 4, 6% | 0.58 |
| **AVA (cm2)** | 0.8 ± 0.2 | 0.8 ± 0.2 | 0.96 |  | 0.8 ± 0.3 | 0.7 ± 0.2 | 0.83 |
| **Mean gradient (mmHg)** | 41 ± 15 | 41 ± 15 | 0.99 |  | 34 ± 12 | 34 ± 12 | 0.99 |
| **Peak Velocity (m/s)** | 3.8 ± 0.7 | 3.8 ± 0.9 | 0.81 |  | 3.9 ± 0.9 | 4 ± 0.9 | 0.89 |
| **Concomitant Valve Disease** | | |  |  |  |  |  |
| **AI Mod or Severe** | 16, 18% | 5, 12% | 0.36 |  | 1, 3% | 7, 10% | 0.19 |
| **MS** | 16, 18% | 5, 12% | 0.37 |  | 7, 19% | 10, 14% | 0.45 |
| **MR Mod or Severe** | 8, 9% | 5, 12% | 0.62 |  | 7, 19% | 13, 18% | 0.86 |
| **TR Mod or Severe** | 6, 7% | 4, 10% | 0.59 |  | 2, 6% | 13, 18% | 0.08 |

Supplemental Table 4: Additional echocardiographic findings for patients with new onset of 30-day LVSVI < 35 ml/m2.

| **Patients with new onset LF (N = 44)** | | |
| --- | --- | --- |
|  | N | % |
| LVEF |  | 56.5% |
| MR (> Moderate) | 2 | 4.5 |
| MS (> Moderate) | 3 | 6.8 |
| TR (> Moderate) | 4 | 9.1 |
| RV dysfunction (Mild+) | 2 | 4.5 |
| E/E' (avg) >14 or E/A > 1.2 | 16 | 36.4 |
